# Person‐Centred Proxy Assessment for People With Advanced Dementia: Transparent Expert Consultation on the Integrated Palliative Care Outcome Scale for Dementia

**DOI:** 10.1002/gps.70264

**Published:** 2026-11-06

**Authors:** Clare Ellis‐Smith, Farina Hodiamont, Marsha Dawkins, India Tunnard, Claudia Bausewein, Jenny T. van der Steen, Maartje S. Klapwijk, Christina Ramsenthaler, Fliss EM. Murtagh, Irene J. Higginson, Catherine J. Evans

**Affiliations:** ^1^ King's College London Cicely Saunders Institute of Palliative Care Policy and Rehabilitation London UK; ^2^ Department of Palliative Medicine, LMU Medizin LMU University Hospital, Ludwig‐Maximilians‐Universität München Munich Germany; ^3^ Guy's and St Thomas' NHS Foundation Trust London UK; ^4^ Wolfson Palliative Care Research Centre Hull York Medical School University of Hull Hull UK; ^5^ Department of Public Health and Primary Care Leiden University Medical Center Leiden the Netherlands; ^6^ Department of Primary and Community Care and Radboudumc Alzheimer Center Radboud university medical center Nijmegen the Netherlands; ^7^ Faculty of Economics and Social Sciences Institute of Sport and Sport Science University of Freiburg Freiburg Germany; ^8^ Department of Health Sciences Institute of Nursing Zurich University of Applied Sciences Winterthur Switzerland; ^9^ King's College London NHS Foundation Trust London UK; ^10^ Sussex Community NHS Foundation Trust Brighton UK

**Keywords:** consensus, dementia, outcome assessment, palliative care, patient‐centered care, proxy, psychometrics, stakeholder participation, symptom assessment

## Abstract

**Objectives:**

The study aimed to strengthen the content validity of a proxy‐reported person‐centred outcome measure, the Integrated Palliative care Outcome Scale for Dementia (IPOS‐Dem), and make recommendations to support its use in routine care to facilitate person‐centred care provision.

**Methods:**

Transparent Expert Consultation using modified nominal group technique in a stakeholder workshop and online consensus survey to generate and prioritise recommendations. Participants included international experts in dementia, palliative care, and geriatrics, and family carers as lived experts. Recommendations from the workshop were thematically analysed and grouped according to priority ranking: ‘retained’, ‘uncertain’, and ‘excluded’. Those categorised ‘uncertain’ were presented in a survey to determine agreement (median) and consensus (interquartile range). For each recommendation, survey participants rated level of agreement from 1 (no agreement) to 9 (high agreement), with option for narrative comments. Descriptive statistics were used for data analysis, and narrative comments analysed thematically.

**Results:**

Twenty‐seven participants attended the workshop generating 179 individual recommendations. Following combination and de‐duplication, 61 recommendations remained. The recommendations formed three themes: ‘The construct of a person‐centred palliative care measure’ (*n* = 13 recommendations), ‘Proxy assessment of subjective symptoms and concerns’ (*n* = 27 recommendations), and ‘Implementation considerations’ (*n* = 21 recommendations). Key recommendations from the workshop concerned the construct of person‐centred assessment specifically how individuals are affected by symptoms and concerns, and use of IPOS‐Dem over time to know the person and monitor symptoms and concerns. Twenty‐one recommendations were grouped as ‘uncertain’ and included in the survey, with 31 survey respondents (74% response rate). Of those recommendations, agreement was highest for the four training recommendations including training on the concept of person‐centred care and training to strengthen reliable proxy assessment (medians ≥ 8).

**Conclusion:**

The findings of this study assert IPOS‐Dem as a measure to support person‐centred assessment and monitoring of distressing symptoms and concerns to improve knowledge of the individual and inform care provision. The term ‘affected by’ is crucial for the validity of IPOS‐Dem as a measure of distressing symptoms but creates challenges for reliable proxy assessment. The recommendations have informed a revised IPOS‐Dem to strengthen its content validity and identified further developments including training and decision‐support tools to enable person‐centred care provision, improve reliability of proxy assessment, and inform care planning.

## Summary

- To ensure content validity, a person‐centred measure of distressing symptoms and concerns must go beyond measuring the presence of symptoms and concerns and instead measure how these affect individuals with dementia.
- Using a comprehensive person‐centred outcome measure in routine care for people with dementia supports greater knowledge of individuals with dementia through systematic person‐centred assessment and monitoring over time.
- Proxy assessment of how symptoms and concerns affect individuals is challenging. Proxies require training to support assessment using non‐verbal signs and observations. Repeated use of a person‐centred outcome measure to gain knowledge of the person over time supports proxy assessment.
- Digital delivery of a person‐centred outcome measure with decision‐support tools and action plans would help provide person‐centred care informed by priorities of individuals and their family carers.

## 1 Introduction

People with dementia live with multiple needs due to dementia and other long‐term conditions common in older age. As dementia progresses, it becomes increasingly challenging to assess a person's needs due to declining verbal communication. Undetected and therefore unaddressed symptoms and concerns cause suffering and are associated with distressed behaviour [1] resulting in increasingly complex care needs [2], family carer burden [3], and increased health and social care use [2, 4]. Palliative care is an approach to maximise comfort and treat distressing symptoms and concerns for people facing life‐threatening or life‐limiting conditions. Core to palliative care is timely, comprehensive person‐centred assessment to ensure distressing symptoms and concerns are treated quickly.

Kitwood's seminal work on personhood in dementia emphasises that people with dementia are unique individuals, and the importance of focussing on individuals' dignity, priorities and wishes [5]. Person‐centred care supports personhood through a holistic approach that puts the person at the centre of their care [6] to address their total care needs [7]. Person‐centred outcome measures are standardised questionnaires which measure the person's own perception of their health status [8]. Used in routine care, they can enhance assessment and management of symptoms and concerns, and improve outcomes of care [8]. While the majority of person‐centred outcome measures are self‐reported [8], proxy‐reported measures are required when individuals are no longer able to self‐report [9, 10]. Measures need to be valid (i.e., measure what they intend to), reliable (i.e., be free from measurement error), acceptable (i.e., relevant to care provision and easy‐to‐use), and feasible (i.e., brief and complement care processes) [11, 12, 13]. There are, however, challenges to the reliability of proxy reporting [9] with known systematic differences between individuals and different proxy groups [14, 15]. Nonetheless, when individuals are no longer able to self‐report, proxy‐reported measures become the only alternative to enable person‐centred assessment.

The Integrated Palliative care Outcome Scale for Dementia (IPOS‐Dem) was developed in 2017 from the IPOS [16], the most recent version of the valid, reliable, and established POS [17]. The IPOS‐Dem is a comprehensive measure to support person‐centred palliative care assessment of people living with dementia and their family carers. Originally developed for use in UK residential care homes (24‐h long term care facilities without onsite registered nurses), the IPOS‐Dem was designed for use by practitioners with no registered clinical qualification to support assessment and review of people with dementia, using non‐clinical terms and/or item descriptors to support accessibility and comprehension. Its rigorous development involved three main steps: (i) review of the literature to identify common symptoms and concerns affecting individuals with dementia, (ii) focus groups with care home practitioners and family carers to develop and evaluate the content validity, and (iii) two rounds of cognitive interviews with care home practitioners to examine its comprehensibility and acceptability [18]. The 31‐item measure starts with an open question about the person's main problems over the past week to understand the individual's priorities. This is followed by questions on how the person has been affected by physical symptoms, including those common in older people living with dementia and other long‐term conditions for example pain and swallowing problems; and questions on psychosocial and existential concerns for example anxiety [18]. Each item is scored from zero to four, with zero indicating no problem and four indicating an overwhelming symptom or concern [18]. Studies have demonstrated that the IPOS‐Dem is acceptable and feasible to use across multiple settings including care homes (long term care facilities), inpatient wards and community settings; with time taken to complete being 5–6 minutes once respondents are familiar with the IPOS‐Dem [19, 20, 21].

The IPOS‐Dem addresses a gap in person‐centred palliative care assessment [22]. Since its development, it has been implemented in services across England [20, 21] and teams globally have commenced translation and cross‐cultural validation, for example in Germany [23], Sweden [24] and China [25]. Its translations and use beyond UK care homes with different respondent groups including people with dementia, family carers and health practitioners in different settings and countries has prompted discussions about how best to use the IPOS‐Dem in routine care to ensure valid and reliable measurement of symptoms and concerns experienced by people with dementia and their families. This study aimed to strengthen the content validity of the IPOS‐Dem for international multi‐setting use and make recommendations to support its use in routine care to facilitate comprehensive person‐centred assessment and management of distressing symptoms and concerns.

## 2 Materials and Methods

### 2.1 Study Design

We convened a Transparent Expert Consultation comprising consensus methods of modified nominal group technique to discuss, generate and prioritise recommendations in an in‐person workshop, followed by an online consensus survey to ascertain level of agreement on priority (Figure 1) [26, 27, 28]. Ethical approval was received on 27 July 2018 from King's College London Research Ethics Committee (LRS‐17/18‐7969).

**FIGURE 1:**
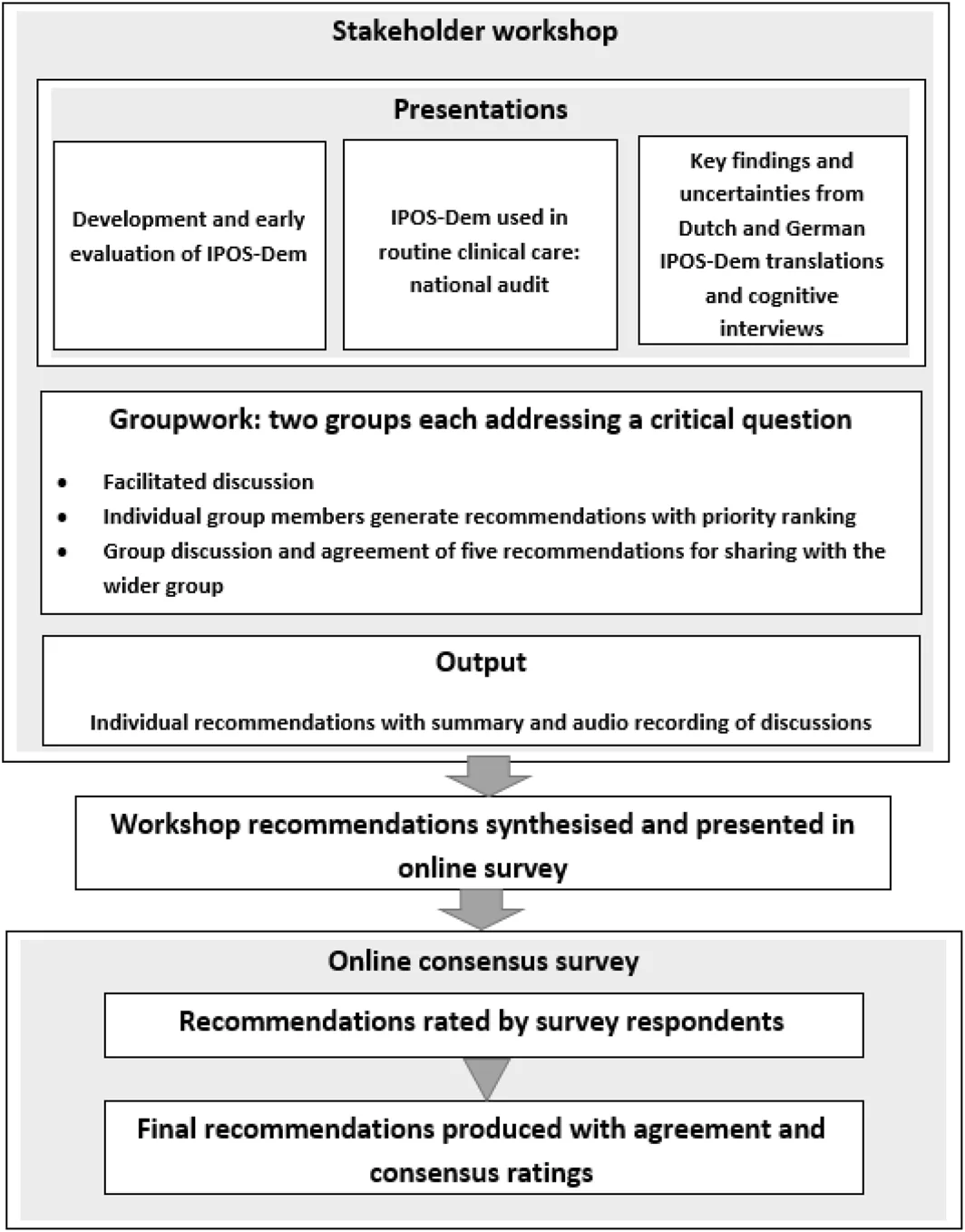
Transparent Expert Consultation: design and methods [20, 21, 22].

### 2.2 Participants and Sampling

Participants were purposively sampled to include international practitioners and researchers with expertise in dementia, palliative care, geriatrics, social care, outcome measurement, and complex interventions; and representatives from dementia charities and family carers as lived experts. Participants were not required to have experience using the IPOS‐Dem. Lived experts were required to have experience in caring for an individual with dementia and/or representing dementia carer groups but not required to have experience of palliative care or using the IPOS‐Dem. Lived experts were reimbursed for travel and time in line with national guidance [29].

Potential participants were identified from existing collaborations/expressions of interest to collaborate and snowballing methods [30]. They included the authors of this study and those who were, or were planning, to translate and cross‐culturally adapt the IPOS‐Dem for use in their own countries. Interested individuals were emailed a personal invitation with a copy of the Participant Information Sheet. Those that indicated interest were sent a pre‐workshop pack which included the Participant Information Sheet, consent form, schedule for the day, a one‐page introduction to POS and IPOS‐Dem, and a copy of the IPOS‐Dem and manual. All participants signed an informed consent form prior to participating in the workshop. Survey participants comprised those attending the workshop, individuals who expressed interest but were unable to attend the workshop, and additional individuals identified in the workshop. Email invitations were sent which included the Participant Information Sheet. Consent was presumed for the survey.

### 2.3 Procedure

#### 2.3.1 Stakeholder Workshop on IPOS‐Dem

A half‐day workshop hosted in London, UK on 17 September 2018 commenced with:

- Presentations on development and early evaluation of the IPOS‐Dem [18, 19], and audit results of its use in routine care in England (CES) [20].
- Key findings and areas of uncertainty from the German translations and cognitive interviews (FH) [23] and Dutch translations (JvdS). These included, for example, use of the phrase, ‘affected by’, use of item descriptors and how they can or should be used in translations, and challenges of proxy assessment.

Two facilitated group discussions followed, addressing questions: (1) how can we maximise valid person‐centred proxy assessment?, and (2) how can we maximise reliable proxy‐reported assessment and minimise missing data? Each group comprised a chair (CES/CJE), facilitator (CB/FEM) and scribe (IT/AJ collaborator) with representation from respective profession/discipline/expertise. Group discussions were audio‐recorded. The modified nominal group technique [26, 27] followed a structured process of:

- Facilitated discussion (20 minutes).
- Individually generating recommendations written on an A4 ‘recommendation sheet’ with indication of priority (1—highest priority to 5—lowest priority) (15 minutes).
- Participants presenting in turn, one recommendation in order of priority with a rationale for their recommendation, until all recommendations were exhausted or time exceeded (20 minutes). Recommendations were written on flip‐chart paper by the scribe.
- Group discussion to agree the top five recommendations generated to share and discuss with the main group to attain an overall understanding of priorities. A PowerPoint slide of top five recommendations was prepared by the scribe (20 minutes).

Supporting Information S1: Appendix 1 details the full workshop schedule.

#### 2.3.2 Workshop Data Analysis

Individual recommendations were pseudonymised and entered into an Excel spreadsheet. The recommendations retained participants' priority level and original language, with minor changes to improve clarity. Verbatim recommendations were used as quotes to illustrate findings. Recommendations were ordered together to identify frequency, combined and de‐duplicated, and synthesised using thematic analysis [31] involving inductive coding to generate themes (CES). Areas of uncertainty and/or contention were discussed with members of the research team (CES/CJE/MD/IT). The recordings were used to enhance understanding of the rationale for recommendations and areas of uncertainty/contention.

To identify recommendations for presentation in the survey, members of the research team (CES/CJE/MD/IT) categorised the recommendations into three groups. Retain: Recommendations with high frequency, high priority (priority level 1 or 2), considered good practice and/or uncontentious. Uncertain: Recommendations with high priority but low frequency, low frequency but high priority, or mixed priority levels. Exclude: Recommendations ranked low priority (priority level 3 or below) or missing priority AND only occurring once with little/no emphasis in the recorded discussion.

#### 2.3.3 Consensus Survey

The survey was piloted by two members of the research team (one international member and one practitioner researcher), with minor refinements to improve clarity. Eligible participants were sent an invitation email/letter with the Participant Information Sheet and link to the online survey or option to complete and return a hard copy. All participants received two reminders. The survey opened on the 11th December 2018 and closed on 9th January 2019.

The survey presented the recommendations for participants to indicate level of agreement of priority from 1 (strongly disagree) to 9 (strongly agree) and provide optional narrative comments [26, 27, 28].

##### 2.3.3.1 Survey Data Analysis

The survey results were exported into an Excel spreadsheet. Descriptive statistics including medians and interquartile ranges (IQR) were used to examine level of agreement and consensus for each recommendation. Levels of agreement and consensus were categorised using established criteria (Table 1) [28, 32]. Free text comments were thematically analysed in Excel to gain further insights into areas of agreement, disagreement, and contention [33].

**TABLE 1:** Categories of levels of agreement and consensus according to medians and interquartile ranges [22, 27].

Median	Interquartile range	Category
≥ 8	< 2	Strong agreement/high consensus
≥ 8	≥ 2	Strong agreement/low consensus
< 8 to > 6	< 2	Moderate agreement/high consensus
< 8 to > 6	≥ 2	Moderate agreement/low consensus
> 4 to ≤ 6	< 2	No agreement/high consensus
> 4 to ≤ 6	≥ 2	No agreement/low consensus
> 2 to ≤ 4	< 2	Moderate disagreement/high consensus
> 2 to ≤ 4	≥ 2	Moderate disagreement/low consensus
≤ 2	< 2	Strong disagreement/high consensus
≤ 2	≥ 2	Strong disagreement/low consensus

#### 2.3.4 Recommendations to Inform Revised Version of IPOS‐Dem

The findings were discussed at POS scientific committee meeting comprising international experts in POS, IPOS and IPOS‐Dem. Each recommendation was presented and discussed with consideration of measurement science, international context, and the wider international POS programme of work to inform refinements to the IPOS‐Dem.

## 3 Results

### 3.1 Participants

Forty‐one eligible individuals were identified and invited to the workshop, of whom 27 attended. Reasons for invitees who expressed interest but were not able to participate included international travel requirements and/or prior commitments. Forty‐two participants were invited to complete the survey including all 27 workshop attendees and those who expressed interest in attending the workshop but were unable to attend. Thirty‐one responded; a response rate of 74%. Participants represented professionals from across the multi‐disciplinary team from palliative care, dementia, geriatric care, and research. Lived experts included those with caring experience (Table 2).

**TABLE 2:** Participant characteristics of the workshop and consensus survey.

	Workshop (*n* = 27)		Consensus survey (*n* = 31)	
	*n*	(%)	*n*	(%)
Profession/role^a^				
Researcher	17	63	17	55
Nurse	5	19	10	32
Medical doctor	8	30	7	23
Allied health professional	2	7	2	7
Health care commissioner	0	0	1	3
Care home practitioner	0	0	1	3
Lived expert representative	6	22	3	10
Charitable organisation representative	0	0	2	7
Other	0	0	1	3
Area of expertise^a^				
Palliative care	14	52	20	65
Research	13	48	15	48
Dementia (older adult mental health/psychiatry)	9	33	12	39
Geriatrics	4	15	8	26
Lived expert (carer expertise)	2	7	2	7
Other	1	4	2	7
Country				
UK	22	82	24	77
Germany	2	7	2	7
Netherlands	2	7	2	7
Sweden	1	4	1	3
Norway	0	0	1	3
Switzerland	0	0	1	3

^a^ Participants could select multiple options.

### 3.2 Stakeholder Workshop

In total, 179 individual recommendations were generated. Following combination and de‐duplication, 61 recommendations remained. The recommendations formed three themes: theme i: ‘The construct of a person‐centred palliative care measure’ (IPOS‐Dem construct) (*n* = 13 recommendations), theme ii: ‘Proxy assessment of subjective symptoms and concerns’ (proxy assessment) (*n* = 27 recommendations), and theme iii: ‘Implementation considerations’ (*n* = 21 recommendations).

Twenty‐four recommendations meeting the criteria of high frequency, high priority and considered good practice and/or not contentious were retained (Supporting Information S2: Appendix 2). Twenty‐one ‘uncertain’ recommendations were presented in the survey (Table 3). Sixteen recommendations were excluded and are detailed in Supporting Information S2: Appendix 2 with reasons. Figure 2 displays the number of recommendations retained, uncertain and excluded by theme.

**FIGURE 2:**
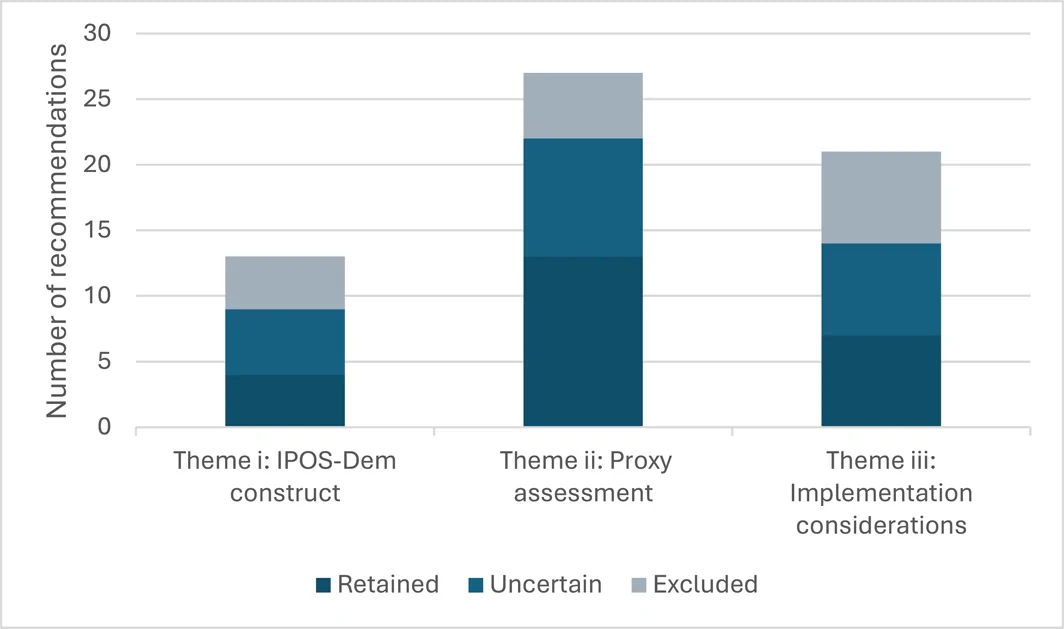
Number of recommendations generated in the workshop that were retained, uncertain and excluded by respective theme.

#### 3.2.1 Theme i: The Construct of a Person‐Centred Palliative Care Measure (IPOS‐Dem Construct)

There were multiple high priority recommendations on the importance of IPOS‐Dem to support comprehensive person‐centred assessment for people with dementia and their families. Recommendations were generated regarding the phrase, ‘… how the person has been affected by each of the following symptoms…’. Retaining the phrase ‘has been affected’ was considered essential to the construct of person‐centred assessment. However, the concept of ‘affected by’ was acknowledged as challenging to comprehension and a threat to reliable assessment.

Please keep 'affected by' somehow as we want to retain the person‐centred perspective that reflects [the] palliative care approach. However, [we] have to recognise this is very hard for carers to do if person does not communicate much….

[CA0017: practitioner researcher]

Recommendations also included the intention of IPOS‐Dem as an assessment to improve awareness of symptoms and concerns, and inform person‐centred care:

Priority should always be to increase awareness [of symptoms and concerns] and so inform care in person‐centred way.

[P0021: lived expert]

The IPOS‐Dem items needed to encompass a holistic approach and be relevant to the care and concerns of people with dementia and their families. Recommendations included items relating to positive aspects of care for the person and family such as ‘what is going well?’, ‘what matters to you?’ and an additional open question asking, ‘what are the main concerns of the family?’. Recommendations in this theme also included the importance of using the IPOS‐Dem to monitor symptoms and concerns, and as a means to get to know the person over time:

Info contained in [the IPOS‐Dem], needs to be available to all concerned with [the] person's care so a clearer identity emerges [and] is gradually built up by all involved.

[P0021: lived expert]

#### 3.2.2 Theme ii: Proxy Assessment of Subjective Symptoms and Concerns

The second theme, and the theme with the most recommendations generated, related to the assessment of subjective symptoms and concerns to inform care. Many of the accepted recommendations pertained to the ‘best’ person to complete IPOS‐Dem, based on their knowledge of the person. Recommendations to maximise reliable person‐centred assessment included proxy reporting by individual(s) who knows the person best (i.e., practitioners, family members), and multiple informants including the person with dementia (where able), family members, and practitioners. There were recommendations about reducing missing responses by removing some symptoms that were particularly challenging to assess for example nausea. However, this conflicted with the construct and intention of IPOS‐Dem as a measure of symptoms and concerns affecting an individual.

The challenge of person‐centred assessment of people with dementia who are unable to verbally express their wishes and concerns was also identified. Recommendations related to the importance of acknowledging the uncertainty and challenges of assessment, and acknowledgement that at times a ‘best guess’ is required. The participants' discussion showed the contention surrounding this, with debate on removing the response option ‘cannot assess’ to ‘force’ proxy respondents to attempt an assessment.

Don’t sacrifice good enough in the pursuit of perfection. It’s most important that there is an assessment completed regardless of the ‘flaws’, ‘glitches’ it contains as it will give a good sense of the needs of the [person with dementia].

[P004: lived expert]

Recommendations also pertained to choice of language and item descriptors to improve comprehension for proxy reporters and clarity regarding the intention of the item, for example, change ‘drowsiness’ to ‘daytime drowsiness’. Practitioner participants, in particular, prioritised the recommendation that agitation is separated from aggression. There was also further discussion that the term, ‘aggression’ should be replaced with ‘distressed behaviour’ to reflect that behavioural changes are frequently a reflection of distress. In the subsequent survey, this recommendation was presented as replacing ‘aggression’ with ‘behavioural difficulties’ (R8).

#### 3.2.3 Theme iii: Implementation Considerations

Accepted recommendations pertained to the use of the IPOS‐Dem in routine care, including using life stories and knowledge of the individual's priorities and preferences when using the IPOS‐Dem; and developing a digital version with action plans to support communication in care. There were multiple high priority recommendations on the imperative of acting upon the IPOS‐Dem scores indicating unmet needs and using a decision support tool to review planned care and treatment.

Develop decision support tool that details action if score at moderate or above e.g. pain, complete detailed pain scale.

[CA0024: practitioner researcher]

Recommendations for implementation included training on the intention of the IPOS‐Dem as a person‐centred assessment, how it should be used in routine care, and how to support assessment of symptoms and concerns using non‐verbal communication signs in people with dementia. Finally, there were recommendations that IPOS‐Dem be used routinely across care settings and stages of dementia. This intended to support getting to know the person over time, especially in the earlier stages when the person may be able to inform the assessment.

All stages of dementia initially completed with the person, accompany through trajectory.

[R0025: researcher]

### 3.3 Consensus Survey

Twenty‐one recommendations categorised as ‘uncertain’ were presented in the survey: theme i: IPOS‐Dem construct (*n* = 5 recommendations), theme ii: Proxy assessment (*n* = 9 recommendations), and theme 3: Implementation considerations (*n* = 7 recommendations). The survey findings indicated strong agreement with eight recommendations, moderate agreement with nine recommendations, no agreement with three recommendations, and disagreement with one recommendation. Consensus was low with only one recommendation achieving strong agreement and high consensus (Table 3, Figure 3).

**TABLE 3:** Table of recommendations included in survey according to themes (*n* = 31 responses).

Recommendations	Median (IQR)^a^
Theme i: The construct of a person‐centred palliative care measure (IPOS‐Dem construct)	
Strong agreement, low consensus	
R12: A second open question should be added to question 1 to incorporate family concerns as follows, ‘what have been the family's priorities over the past week?’	8 (5–9)
R15: Remove item, ‘what was the person's last weight and date s/he was last weighted?’ as this does not need to be completed in IPOS‐Dem when it is recorded as part of routine practice. Adding it creates burden for staff to fill in when it is already recorded elsewhere	8 (5–9)
Moderate agreement, low consensus	
R7: IPOS‐Dem should not just be about problems. The first IPOS‐Dem question should encompass both positive and negative aspects as follows, ‘what are the person's priorities of care over the past week?’ $	7 (6–9)
R14: Remove item ‘can s/he enjoy activities appropriate for his/her level of interest and abilities?’ as it overlaps with item ‘lost interest in things s/he would normally enjoy’	7 (5.5–9)
No agreement, low consensus	
R13: Nausea is an uncommon symptom experienced by people with dementia and therefore has less clinical relevance. It should be removed	5 (4.5–7.5)
Theme ii: Proxy assessment of subjective symptoms and concerns (proxy assessment)	
Strong agreement, low consensus	
R11: Item ‘have all practical problems been addressed? e.g. hearing aids, foot care, glasses, diet’ is changed to ‘have all practical problems been considered? e.g. hearing aids, foot care glasses, diet.’	8 (6–9)
R16: Remove item ‘has his/her family had enough information as wanted?’ and replace with ‘are the family aware of the current situation or recent changes’ taken from another tool	8 (6–9)
Moderate agreement, low consensus	
R10: Item ‘Poor appetite’ is changed to ‘change of eating behaviour’ as this is easier to assess objectively	7 (6–9)
R6: To support completion and acknowledge the subjective judgement of assessment of symptoms and concerns, the phrase, ‘do you think?’ should be incorporated into IPOS‐Dem questions e.g. ‘Please select one box that best describes how you think the person has been affected by each of the following symptoms over the past week …’	7 (6–8)
R4: In the assessment of a person with dementia, the person/people completing IPOS‐Dem may be required to make a subjective judgement. A ‘best guess’ is preferable to no response even if there is doubt	7 (5–8)
R8: Replace the term ‘aggression’ with ‘behavioural difficulties’	7 (5–8)
R3: In the assessment of a person with dementia, any assessment is better than no assessment. This is the case even if the person/people completing IPOS‐Dem do not know the person with dementia	7 (4–7)
No agreement, low consensus	
R9: Spiritual concerns is assessed by the item ‘at peace’.	6 (3.5–8)
Moderate disagreement, low consensus	
R5: The response option ‘cannot assess’ should be removed	3 (1.5–5)
Theme iii: Implementation considerations	
Strong agreement, high consensus	
R18: IPOS‐Dem manual and training should encompass the concept of person‐centred care for people with dementia	9 (8–9)
Strong agreement, low consensus	
R21: IPOS‐Dem manual and training should include detail on who should complete it and why	9 (7–9)
R20: IPOS‐Dem manual and training should include item descriptors (e.g. description of body language/facial expression) to support assessment of challenging symptoms and concerns	8 (7–9)
R19: Response options of IPOS‐Dem (e.g. mild, moderate, severe, overwhelming) should be defined in the manual and training, i.e. there should be a description of how each severity rating presents $	8 (6.25–9)
Moderate agreement, low consensus	
R17: There are two items relating to family, including ‘have any of his/her family been anxious or worried about the person?’ currently grouped with psychosocial items, and ‘has his/her family had as much information as wanted?’ currently grouped with practical matters. The family items should be moved so that they are grouped together	7 (5.5–9)
R1: IPOS‐Dem should be used routinely for all people with dementia and their families across all dementia stages and care settings	7 (5–9)
No agreement, low consensus	
R2: IPOS‐Dem is developed for people with dementia and multi‐morbidities. It should therefore be used for all people living in care homes including those without dementia or cognitive impairment	5 (3–7.5)

*Note:* $—*n* = 30.

^a^ IQR—interquartile range.

**FIGURE 3:**
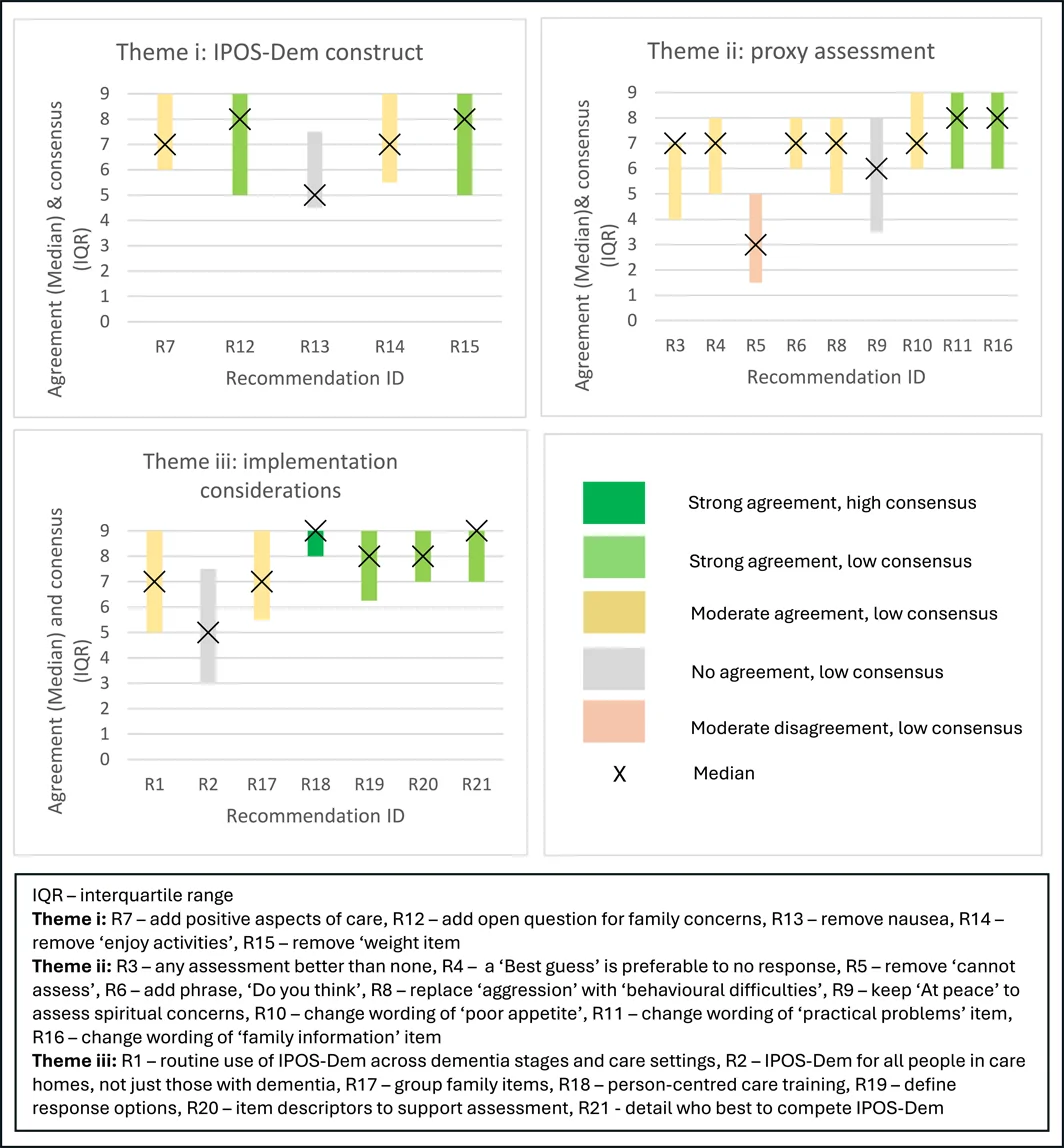
Agreement and consensus for each recommendation by theme.

#### 3.3.1 Theme i: The Construct of a Person‐Centred Palliative Care Measure (IPOS‐Dem Construct)

Participants strongly agreed to the addition of an open question of family concerns (R12) and to remove the item asking about weight (R15). They moderately agreed with the recommendations, ‘IPOS‐Dem should not just be about problems and should incorporate positive aspects of care’ (R7) and to remove the item, ‘enjoying activities’ (R14). Participants neither agreed nor disagreed with the recommendation to remove ‘nausea’ (R13), with free text comments indicated conflicting opinion about removing nausea. Some participants cited the challenge of accurate assessment as a reason to remove nausea. Others stated that it is an important, relevant and distressing symptom that may be a side effect of medication and should therefore be included as part of a comprehensive assessment.

I think it is an assumption that it is experienced less often as we cannot judge it.

[CR003: practitioner researcher]

#### 3.3.2 Theme ii: Proxy assessment of Subjective Symptoms and Concerns (Proxy Assessment)

There was strong agreement for two recommendations to change the wording for the items pertaining to practical problems (R11) and family information (R16). Recommendations with moderate agreement included: ‘Any assessment is better than no assessment even if the respondent does not know the individual’ (R3), ‘A best guess is better than no response’ (R4), and the addition the phrase ‘Do you think’ to acknowledge subjective judgement in assessment (R6). Participants moderately disagreed with the removal of the response option ‘cannot assess’ (R5). Free text supporting removal of ‘cannot assess’ included statements that respondents might select this option without attempting assessment. Conversely, there was concern that removing ‘cannot assess’ would force respondents to guess resulting in inaccurate assessment:

This is a tricky one—…, gives an option for people not to “make stuff up”. However, does also give people a get out not to try to find information. I am hopelessly on the fence for this one, as can see it both ways—sorry.

[C0018: practitioner]

Other recommendations with moderate agreement pertained to changing language and wording of items (R8, R10). Participants neither agreed nor disagreed with R9—Changing the wording of ‘At peace’. Free text indicated concerns that this term does not evidently assess spiritual concerns and/or is ambiguous in meaning, for example that the ‘at peace’ suggests that the person has died [CR0014: practitioner researcher].

#### 3.3.3 Theme iii: Implementation Considerations

This theme had the strongest agreement, particularly with recommendations for training. Participants agreed with four recommendations, one of these with high consensus, ‘Training on the concept of person‐centred care for people with dementia’ (R18). The remaining recommendations with strong agreement/low consensus were ‘Providing detail on who should complete IPOS‐Dem’ (R21), ‘Training/support to assess symptoms in people unable to communicate verbally’ (R20) and ‘Providing definitions of response options for example ‘severe’ (R19). Free text indicated that the reason for low consensus was concern that specifying who should complete IPOS‐Dem would risk non‐completion and concern that a manual may lose acceptability if it is too long:

It is nice to have, although I feel if the Manual gets too big, it will shy away the nursing assistant staff to complete as they may feel overwhelmed.

(CR0034: practitioner researcher)

Recommendations with lower agreement related to the settings and populations that IPOS‐Dem should be used with. Those with agreement were ‘IPOS‐Dem should be used routinely across all stages of dementia and settings’ (R1) and ‘Family items should be grouped together’ (R17). Participants neither agreed nor disagreed with the recommendation that in care homes, IPOS‐Dem should be used with all residents not just those with dementia (R2).

*Y*es because I think we need to recognise and start discussing dementia as palliative from the outset across all services and capture this routines via measures like IPOS‐Dem across the trajectory and it seems to me to be the best outcome measure to do so.

[CR0013: practitioner]

Participants who disagreed with these recommendations tended to agree with the principles but suggested more research was required (R1 and R2), with consideration of feasibility for use with all residents in care homes (R1), and validity of IPOS‐Dem for people without dementia (R2).

This is a very 'black and white' statement; I therefore find it had to agree with. However, I would support the use of IPOS‐Dem widely to support care once it has been validated (and been shown to be psychometrically and clinically robust and capturing what is important to the individuals concerned).

[CR0017: practitioner researcher]

### 3.4 Recommendations to Inform Next Version of the IPOS‐Dem

The findings were discussed at an in‐person POS scientific meeting on 27 February 2019 comprising international experts in POS, IPOS and IPOS‐Dem (including author attendees: CES, CJE, IJH, MD, JvdS, FH, CB, IT). Recommendations informed refinement of IPOS‐Dem to develop a newer internationally relevant version reflecting the identified construct of IPOS‐Dem (Supporting Information S3: Appendix 3).

## 4 Discussion

Three themes were identified from the workshop: ‘the construct of a person‐centred palliative care measure’, ‘Proxy assessment of subjective symptoms and concerns’, and ‘Implementation considerations’. Participants generated recommendations that the IPOS‐Dem is a person‐centred palliative care measure of individuals with dementia and their family carers. As such, it must be comprehensive and should be used to inform care provision through increased knowledge of the person over time by identifying and monitoring to what extent individuals are affected by symptoms and concerns. To facilitate this, individuals with dementia whenever possible, should be consulted and involved in the completion of the IPOS‐Dem; and proxies may include multiple individuals who know the person well. Training is required on person‐centred care, the intention and construct of IPOS‐Dem, and who is best placed to complete the IPOS‐Dem. Recommendations that were retained from the workshop due to high frequency and high priority included development of a digital IPOS‐Dem incorporating decision‐support tools and a clear action plan to support management of identified symptoms and concerns.

This study strengthens the existing evidence that systematic use of person‐centred outcome measures can support a person‐centred palliative care approach [19, 34, 35, 36]. The recommendations both confirm and inform the content validity of IPOS‐Dem as a comprehensive palliative care measure of symptoms and concerns that affect individuals and their family carers. This means it is not just a measure of the presence and severity of symptoms and concerns but that it identifies those that cause the most distress and are therefore the highest priorities for care. Nonetheless, the importance of practitioner training on the concept of person‐centred care and the intention of IPOS‐Dem to support person‐centred care is considered essential, demonstrating that, for practitioners not trained in dementia or palliative care, a person‐centred approach may be unfamiliar and the intention of IPOS‐Dem not immediately obvious.

Proxy assessment of subjective symptoms and concerns is consistently identified as challenging [20, 37]. It is based on the knowledge and observations of the person over time. This means that those with different knowledge of the person (e.g., family carers versus practitioners) or those who observe individuals in different circumstances (e.g., day versus night shifts) may have systematically different responses [15, 38, 39, 40]. Given the challenges of reliable proxy assessment, particularly of subjective concerns, caution is recommended [38]. ‘Perfect’ proxy‐reported measures may be impossible to achieve, but this should not preclude person‐centred outcome measurement to inform care. Though possible, it is less likely that a lack of strong reliability will result in harm to the person with dementia compared to not using a proxy measure, where symptoms or concerns may still be under‐ or over‐estimated, or worse, not considered at all. Training may help improve the reliability of proxy‐reported measures with our participants having strong agreement on all three recommendations on training to improve reliability of IPOS‐Dem.

This study has a number of important research and care implications. First, is the development of training and resources for proxy informants to understand the concept and intention of person‐centred care and to support reliable assessment. Second, the development of a digital version of IPOS‐Dem with decision‐support tools and action plans linked to address identified problems with a planned date for a repeat IPOS‐Dem assessment. Third, refinements were made to IPOS‐Dem. These included, for example, the addition of an open question, ‘What have been the family's, or those closest to them, main concerns about the person in the last week?’, and removing items, ‘enjoying activities’, ‘weight’, ‘family information’, and ‘aggression’. Where there was uncertainty or lack of strong evidence either way, the committee advised that translations explore alternative suggestions and that these items are scrutinised when conducting psychometric evaluations (Supporting Informatin S3: Appendix 3). IPOS‐Dem has been revised to strengthen the content validity and is freely available on the POS website [41]. Fourth, is the use of IPOS‐Dem in wider settings and populations. An accepted recommendation was that the IPOS‐Dem should be used throughout the dementia trajectory to enable a good knowledge of the person as dementia progresses. Using IPOS‐Dem across settings and developing a self‐report version may support comprehensive assessment of people with dementia earlier in the disease trajectory who are still able to self‐report but requires further research. Finally, further testing of IPOS‐Dem is required to evaluate possible risk of harm of proxy reported measurement in routine care and psychometric testing of all versions with different respondents.

There were strengths and limitations to this study. This research was conducted in 2018 before Covid‐19 which raises questions as to the relevance of the findings post‐pandemic. However, the pandemic and research conducted during this time highlighted the imperative of comprehensive palliative care assessment [42] to ensure rapid identification and response to distressing concerns [43, 44], and the requirement to prioritise personhood and comfort for people with dementia throughout the trajectory [45]. Since the pandemic services have reported the benefit of using the IPOS‐Dem to assess palliative care symptoms and concerns in people with dementia [21]. Together, this has highlighted the ongoing relevance and importance of these findings. Meanwhile, there has been a growing international programme to develop the science of IPOS‐Dem as a complex intervention to improve care and outcomes for people with dementia and family carers, its implementation and use across settings, and the ongoing translations and cross‐cultural adaptations [23, 24, 25, 36]. This has highlighted the requirement to define the construct and intention of IPOS‐Dem ensuring conceptual equivalence of translations. This indicates the continued importance and relevance of this work.

We recruited multiple professions and disciplines with dementia expertise including those with lived experience, with international representation which provided diverse perspectives. However, the majority of the participants were from the UK and Western Europe limiting applicability to non‐Western cultures and contexts. Current and future work translates and develops the IPOS‐Dem for non‐European countries including for example, China [25]. A limitation was that most analysis and synthesis was conducted by one researcher and reviewed with the wider research team. The consensus survey comprised one round, limiting the refinement of the recommendations and exploration of contentious areas.

## 5 Conclusion

The study findings assert the IPOS‐Dem as a person‐centred palliative care measure of symptoms and concerns affecting individuals with dementia. The term ‘affected by’ is essential for its validity but creates additional challenges to reliable proxy assessment. The recommendations have informed further developments. These include (i) training and resources to support person‐centred care provision and how the IPOS‐Dem may enable this, (ii) training and resources to support reliable proxy assessment, (iii) development of action plans with decision‐support tools to support management of identified symptoms and concerns; and (iv) further feasibility testing in care of people with dementia across settings [46, 47].

## Conflicts of Interest

The authors declare no conflicts of interest.

## Supporting information


Supporting Information S1

Supporting Information S2

Supporting Information S3

## Data Availability

The data that support the findings of this study are available on request from the corresponding author. The data are not publicly available due to privacy or ethical restrictions.
