# Supplementary material for: Person‐Centred Proxy Assessment for People With Advanced Dementia: Transparent Expert Consultation on the Integrated Palliative Care Outcome Scale for Dementia
Source: Int J Geriatr Psychiatry. 2026 Nov 6;41(11):e70264. doi: 10.1002/gps.70264 (PMC13743913; doi:10.1002/gps.70264)
Supplement: Supplementary file 1 [file GPS-41-e70264-s001.docx]

# Appendix 2: Workshop recommendations not included in the survey

Stakeholder workshop recommendations accepted due to high frequency, high priority, not contentious and/or considered good practice

Theme i: The construct of a person-centred palliative care measure (IPOS-Dem construct)

1. IPOS-Dem should be of clear relevance to routine care and brief
2. The intention of IPOS-Dem is to gain knowledge of the person over time
3. The intention of IPOS-Dem is to monitor and scores should be considered in context of previous scores
4. Retain ‘affected by’ as this reflects the person-centred perspective of palliative care approach

Theme ii: Proxy assessment of subjective symptoms and concerns (proxy assessment)

1. Include a question on who was involved in completing IPOS-Dem today (multiple may be selected): person with dementia, family member, keyworker, health care professional (please state), care team
2. Wherever possible and to ensure consistency, the person/people completing IPOS-Dem should know the best person best
3. Wherever possible, IPOS-Dem should be informed by multiple people who know the person
4. IPOS-Dem scores of 3 and above should prompt further detailed assessment using, for example, recommended pain assessment tools with a link and further observations
5. Person with dementia should be involved in reporting in the early stages of dementia so that a good knowledge of the person is obtained as dementia progresses
6. Wherever possible, the person with dementia should be consulted when IPOS-Dem is completed
7. Assess whether person with dementia can reliably self-report. If yes, complete IPOS-Dem with them. If no, but can still verbally express themselves, then person/people completing IPOS-Dem should consult person with dementia
8. Ensure that information is gathered from families prior to completing IPOS-Dem, and this information is used in reviews, care planning and handovers
9. Be consistent with descriptions in brackets, sometimes these refer to explanations and sometimes examples. If example, state that it is an example.
10. Change ‘*drowsiness’* to *‘daytime drowsiness’*
11. Change *‘sleepiness’* to *‘night-time sleeping problem’*
12. Separate aggression from agitation
13. There should be flexibility to use appropriate terms in translations and cross-cultural adaptions

Theme iii: Implementation considerations

1. There should be a clear action plan at the end of IPOS-Dem including what needs to be done to address any identified problems, and when a repeat IPOS-Dem is indicated
2. Wherever possible, information about the person, including life story, priorities and preferences, and normal presentation should be known to the person/people completing IPOS-Dem
3. Develop an electronic version with support to assess the person, and action plans based on scores, which can also support communication
4. Include a question about whether this IPOS-Dem assessment is routine or because there has been a change in presentation
5. Mini-Mental State Examination (MMSE) (or other cognitive testing/staging) is not appropriate to use with IPOS-Dem
6. Clear intention of and construct of IPOS-Dem must be provided in IPOS-Dem manual and training
7. Keep time frame as past week

Stakeholder workshop recommendations from workshop not included in survey due to low frequency (once only) and low priority (priority 3-5) with reasons

| Recommendation | Reason for non-inclusion |
| --- | --- |
| Theme i: The construct of a person-centred palliative care measure (IPOS-Dem construct) | |
| IPOS-Dem should be used as a checklist of symptoms/concerns for carers | Recommended once - priority 3 |
| Replace drowsiness and lost interest with apathy to improve relevance to people with dementia and carers | Recommended once - priority 5 |
| The intention of IPOS-Dem is to gain multiple perspectives from people involved in care to meet care needs, and consistency therefore matters less | Recommended once, priority missing |
| To improve person-centredness, move psychosocial concerns before physical symptoms | Recommended once – priority missing |
| Subtheme i: Subjective assessment | |
| Change shortness of breath to difficulties in breathing | Recommended once - priority 4 |
| Swallowing: Change descriptor to 'coughing on food/drink' | Recommended once - priority 4 |
| It is important for care home staff to understand family relationship with person with dementia (a positive or negative relationship) may result in over or under-estimates | Recommended once - priority missing |
| Contract dental problem and mouth problems into one - this can then be followed up in oral health assessment for care homes (which we have) | Recommended once - priority missing |
| State what information this refers to, e.g. 'Has his/her family had enough information about care and treatment, and what to expect' | Recommended once - priority missing |
| Subtheme iii: Implementation considerations | |
| Training beyond IPOS-Dem in what could be contributing to behaviours | Recommended once - priority 3 |
| Provide examples or vignettes to inform assessment | Recommended once - priority 3 |
| Provide training for use with all stages of dementia | Recommended once - priority 4; and incorporated into other recommendations |
| Where appropriate, IPOS-Dem should be used a trigger for ACP discussions, particularly when burdensome symptoms are identified | Recommended once - priority 4 |
| Define the target population well and not by place of care which may vary internationally | Recommended once -priority 5 |
| Provide alternative if no family | Recommended once -priority 5 |
| Add a question about whether the person is able to ask for help | Recommended once - priority missing |
