# Supplementary material for: Person‐Centred Proxy Assessment for People With Advanced Dementia: Transparent Expert Consultation on the Integrated Palliative Care Outcome Scale for Dementia
Source: Int J Geriatr Psychiatry. 2026 Nov 6;41(11):e70264. doi: 10.1002/gps.70264 (PMC13743913; doi:10.1002/gps.70264)
Supplement: Supplementary file 2 [file GPS-41-e70264-s002.docx]

### **Appendix 3: Decisions for the next version of IPOS-Dem & proposed further research**

Table 1: Decisions for the next version of IPOS-Dem

| **Recommendation** | **Discussion** | **Outcome** |
| --- | --- | --- |
| Overarching theme: Person-centred assessment: construct and relevance to care | | |
| R7: IPOS-Dem should not just be about problems. The first IPOS-Dem question should encompass both positive and negative aspects as follows, ‘What are the person’s priorities of care over the past week?’  Moderate agreement, low consensus | Discussion of the use of “concerns/priorities/problems”. | Continued use of the word problem (for patients) but use of concerns for family.  Split this question into two items:  1a: What have been the person’s main problems over the past week?  1b: What have been the family’s, or those close to them, main concerns about the person over the past week? |
| R12: A second open question should be added to question 1 to incorporate family concerns as follows, ‘What have been the family’s priorities over the past week?’  Strong agreement, low consensus |  |  |
| R13: Nausea is an uncommon symptom experienced by people with dementia and therefore has less clinical relevance. It should be removed  No agreement, low consensus | The German version has retained nausea. | Retain nausea to ensure comprehensive assessment with recognition of the that it is difficult to assess. |
| R14: Remove item ‘Can s/he enjoy activities appropriate for his/her level of interest and abilities?’ as it overlaps with item ‘Lost interest in things s/he would normally enjoy’  Moderate agreement, low consensus | Too much overlap between the items. | Remove item, ‘Can s/he enjoy activities?’ in line with the German version. |
| R15: Remove item, ‘What was the person’s last weight and date s/he was last weighted?’ as this does not need to be completed in IPOS-Dem when it is recorded as part of routine practice. Adding it creates burden for staff to fill in when it is already recorded elsewhere  Strong agreement, low consensus |  | Remove weight item to reduce burden and creates duplication in care processes. |
| Subtheme i: Assessment of subjective symptoms and concerns | | |
| R5: The response option ‘Cannot assess’ should be removed  Moderate disagreement, low consensus | Discussion of the importance of retaining ‘cannot assess’ as it provides data, i.e. useful to learn number of assessments required before assessment can take place. Additionally, ‘cannot assess’ may allow for IPOS-Dem to be administered to those without dementia. If an item is not relevant staff should select ‘not at all’ and this should be added to manual.  Discussion around the use of ‘not relevant’. Group agree to add a box below ‘not at all’ box in family items for ‘not relevant, no family’. | Retain, ‘Cannot assess’  If an item is not relevant staff should select ‘not at all’ and this should be added to manual.  To add a box below ‘not at all’ box in family items for ‘not relevant, no family’. |
| R6: To support completion and acknowledge the subjective judgement of assessment of symptoms and concerns, the phrase, ‘Do you think?’ should be incorporated into IPOS-Dem questions e.g. ‘Please select one box that best describes how you think the person has been affected by each of the following symptoms over the past week …’  Moderate agreement, low consensus | Concern that respondents may be reluctant to provide their own judgement. Germany have added “try to imagine…” and brief explanations to items. | No changes to be made to the English version and to learn from the results of the German version. A note to promote confidence could also be added to the manual. |
| Subtheme ii: Comprehension of terms | | |
| R8: Replace the term ‘aggression’ with ‘behavioural difficulties’  Moderate agreement, low consensus | Opportunity to review this item once factor analyses have been conducted for both English and German versions | Remove ‘aggression’ from the English version of IPOS-Dem. Retain in the German version. |
| R9: Spiritual concerns is assessed by the item ‘at peace’. Do you agree with this term? If you disagree, please suggest alternatives.  No agreement, low consensus | Both German and Dutch professionals have found this easy to understand. No viable alternative could be identified. | Retain ‘at peace’ without change. |
| R10: Item ‘Poor appetite’ is changed to ‘Change of eating behaviour’ as this is easier to assess objectively  Moderate agreement, no consensus |  | Retain ‘poor appetite’ without change. |
| R11: Item ‘Have all practical problems been addressed? e.g. hearing aids, foot care, glasses, diet’ is changed to ‘Have all practical problems been considered? e.g. heating aids, foot care glasses, diet.’  Strong agreement, low consensus | Lack of clarity surrounding ‘been addressed’, it is not clear if action (if possible) has been taken. | Add detail in the IPOS-Dem manual about how to complete this item. |
| R16: Remove item ‘Has his/her family had enough information as wanted?’ and replace with ‘Are the family aware of the current situation or recent changes’ taken from another tool  Strong agreement, low consensus | This item has been removed from the German version and replaced with a question on unmet family needs. The group agree to remove but not to replace. | Remove item ‘*Has his/her family had enough information as wanted?’* |
| Subtheme iv: Settings and populations | | |
| R17: There are two items relating to family, including ‘Have any of his/her family been anxious or worried about the person?’ currently grouped with psychosocial items, and ‘Has his/her family had as much information as wanted?’ currently grouped with practical matters. The family items should be moved so that they are grouped together  Moderate agreement, low consensus |  | ‘Family information’ item is to be removed (R16), therefore this recommendation is no longer applicable. |
|  |  |  |

Table 2: Proposed further research

| **Recommendation** | **Proposed future research** | **Rationale** |
| --- | --- | --- |
| Subtheme i: Assessment of subjective symptoms and concerns | | |
| R3: In the assessment of a person with dementia, any assessment is better than no assessment. This is the case even if the person/people completing IPOS-Dem do not know the person with dementia  Moderate agreement, low consensus | - Training developed to support proxy assessment - Instructions that person who knows them best should complete and use all available information - Test for risk of harm - Test feasibility - Psychometric evaluation | It is important to acknowledge that there are challenges to proxy assessment; and that subjective judgement is required.  It is important that an assessment is attempted but caution about risk of harm. It is important to indicate level of certainty. |
| R4: In the assessment of a person with dementia, the person/people completing IPOS-Dem may be required to make a subjective judgement. A ‘best guess’ is preferable to no response even if there is doubt  Moderate agreement, low consensus | - Acknowledge no gold standard for proxy assessment - Option of indicating uncertainty - Training for proxy assessment - Instructions that person who knows them best should complete and use all available information - Use of ‘cannot assess’ | There is no gold standard for proxy assessment, and acknowledgement that subjective judgement is required.  However, there may be a risk of harm if ‘best guess’. |
| Subtheme iii: Training and implementation requirements | | |
| R18: IPOS-Dem manual and training should encompass the concept of person-centred care for people with dementia  Strong agreement, high consensus | Develop training on person-centred care | Evidence of varying degrees of knowledge of person-centred care across countries which may be cultural.  PPI and clinicians considered person-centred care as essential practice. Other participants reported person-centred care being an unfamiliar concept.  One participant reported that professional carers should already have a good understanding of person-centred care and that too much detail for manual. |
| R19: Response options of IPOS-Dem (e.g. mild, moderate, severe, overwhelming) should be defined in the manual and training, i.e. there should be a description of how each severity rating presents  Strong agreement, low consensus | Acceptable (easy to use), and psychometrically sound severity descriptors | Used in other measures including STAS. One participant also stated useful for HoNOS65+ |
| R20: IPOS-Dem manual and training should include item descriptors (e.g. description of body language/facial expression) to support assessment of challenging symptoms and concerns  Strong agreement, low consensus | Develop item descriptors | Agreement that this would support reliable assessment of people who are not able to verbally communicate.  One respondent (PPI) expressed concern that this not reliable method of assessment |
| R21: IPOS-Dem manual and training should include detail on who should complete it and why  Strong agreement, low consensus | The original manual includes details on the purpose of IPOS-Dem, who is best to complete it and suggested frequency. This can be further developed based on emerging evidence. |  |
| Subtheme iv: Settings and populations | | |
| R1: IPOS-Dem should be used routinely for all people with dementia and their families across all dementia stages and care settings  Moderate agreement, low consensus | Participants endorsed this recommendation but recommended that more evidence is required:   - Person versus proxy version - Psychometrically robust in all populations and care settings - Relevant to people with mild dementia - Training for respondents across settings - Feasibility testing for routine use | This recommendation addresses a gap in dementia specific measures  It recognises palliative care throughout the disease trajectory  Participants disagreeing expressed concern that not relevant to mild dementia, developed for proxy-reporting so not suitable for those who can self-report.  More evidence required before it is used across trajectory and across settings, especially regarding training requirements and feasibility |
| R2: IPOS-Dem is developed for people with dementia and multi-morbidities. It should therefore be used for all people living in care homes including those without dementia or cognitive impairment  No agreement, low consensus | Further evidence required to assess whether IPOS-Dem is relevant, and psychometrically sound for all residents in care homes | Participants supporting this recommendation stated it would support implementation and that there is high prevalence of cognitive impairment in care homes. Participants supporting this also advised that it addresses an important gap of comprehensive assessment of people living in care homes.  Those disagreeing felt that more appropriate tools e.g. IPOS and that proxy-reported measure should not be used for people who can self-report |
