# Supplementary material for: Person‐Centred Proxy Assessment for People With Advanced Dementia: Transparent Expert Consultation on the Integrated Palliative Care Outcome Scale for Dementia
Source: Int J Geriatr Psychiatry. 2026 Nov 6;41(11):e70264. doi: 10.1002/gps.70264 (PMC13743913; doi:10.1002/gps.70264)
Supplement: Supplementary file 3 [file GPS-41-e70264-s003.docx]

### **Appendix 1: Workshop schedule**

# OPTCare Dem: Optimising care in dementia by developing a novel international measure to aid assessment and care

## Stakeholder Consultation Workshop

## Cicely Saunders Institute, Monday 17^th^ September 12:30 – 17:00

| **Time** | **Item** | **Chair / lead** |
| --- | --- | --- |
| 12.30-13:15 | Registration, consent & lunch | |
| 13:15-13:30 | Welcome and introduction to the POS family of measures | *Dr Catherine Evans* |
| 13.30-14:00 | Development of IPOS-Dem and presentation of IPOS-Dem across clinical settings. | *Dr Clare Ellis-Smith* |
| ***IPOS-Dem translation findings*** | | |
| 14:00-14:15 | Translation of German IPOS-Dem: presentation of results | *Farina Hodiamont* |
| 14.15-14:30 | Translation of Dutch IPOS-Dem: presentation of results | *Dr Jenny van der Steen* |
| 14.30-15.00 | Discussion and presentation of critical questions | *Dr Catherine Evans and Dr Clare Ellis-Smith* |
| 15.00-15.15 | **Coffee break** | |
| 15.15-16.30 | Nominal groups to discuss:  Question 1: How can we maximise valid person-centred, proxy assessment?  Question 2: How can we maximise reliable proxy-reported assessment, and minimise missing data? | *Facilitators and scribes* |
| 16.30-17.00 | Feedback from small groups to whole group and closing remarks | *Dr Catherine Evans and Dr Clare Ellis-Smith* |
